# A rare bacteremia caused by *Fannyhessea vaginae* in a pregnant woman: case report and literature review

**DOI:** 10.3389/fcimb.2023.1278921

**Published:** 2023-12-08

**Authors:** Peng Liu, Lina Wang, Rongguo Li, Xiaodi Chen

**Affiliations:** Department of Clinical Laboratory, Jinan Maternity and Child Care Hospital Affiliated to Shandong First Medical University, Jinan, Shandong, China

**Keywords:** anaerobic bloodstream infections, bacteremia, case report, *Fannyhessea vaginae*, literature review

## Abstract

Bloodstream infection caused by anaerobic microorganisms continues to be associated with a high mortality risk, necessitating a rapid diagnosis and an appropriate treatment. As an anaerobic gram-positive organism associated with vaginal infections, *Fannyhessea vaginae* is a rare cause of invasive infections. In this case, a 32-year-old pregnant woman with bacterial vaginosis presented with bacteremia. The microbiological analysis of the blood cultures identified *F. vaginae*. The patient was treated empirically with 5 days of cefoperazone/sulbactam and recovered well. Here, we provide a review of the literature on *F. vaginae* infections, and the reported cases demonstrate the need for awareness of the different anaerobic species found in the vaginal tract and adaptation of empirical therapies, especially in pregnant women.

## 1 Introduction

Anaerobes are the dominant organisms of the normal human microbiome. They inhabit mucosal membranes such as those in the female reproductive tract, gastrointestinal system, and oral cavity. Generally, these organisms play a crucial role in sustaining normal homeostasis in the human body. However, they can also serve as pathogens that cause invasive infections in human populations (Watanabe et al., 2021). Anaerobic bloodstream infections are responsible for up to 20% of bacteremic episodes with a high mortality rate, even higher in patients who are of advanced age and lack appropriate treatment (Dien Bard et al., 2020).

*Fannyhessea vaginae*, previously known as *Atopobium vaginae*, is a strict anaerobe first isolated from the vaginal flora of a healthy Swedish woman in 1999 (Jovita et al., 1999). In 2018, it was reclassified as *F. vaginae* (Nouioui et al., 2018). *F. vaginae* is a Gram-positive, elliptical- or rod-shaped coccobacillus that can appear as single elements, pairs, clumps, or short chains and is a part of the human vaginal microbiome. Many studies have emphasized that *F. vaginae* plays an important role in the pathophysiology of vaginal diseases (Mendling et al., 2019). *F. vaginae* is able to incorporate into *Gardnerella vaginalis* biofilms, a crucial marker of bacterial vaginosis (Castro et al., 2021). It has also been determined that high vaginal loads of *F. vaginae* in conjunction with *Gardnerella* spp. is linked to late miscarriage and preterm birth (Bretelle et al., 2015). Although *F. vaginae* can be detected in the normal vaginal microbiome (8%–25%), it is more prevalent in patients with bacterial vaginosis (50%–96%) (Mendling et al., 2019). However, *F. vaginae* is an uncommon cause of invasive infections.

Here, we describe a rare case of bacteremia caused by *F. vaginae* in a pregnant woman with bacterial vaginosis and hypothesize that an ascending infection of *F. vaginae* in the vagina caused this woman’s bacteremia. We also provide a review of previously published cases related to *F. vaginae* infections. The reported cases demonstrate that if a patient is febrile and exhibits symptoms of bloodstream infection, anaerobic species that are prevalent in the vaginal tract should be considered, especially for pregnant women with vaginal infections

## 2 Case report

### 2.1 Case description

The patient, a 32-year-old woman with uterine fibroids and resistant hypertension, was admitted for the delivery of her second child at a gestational age of 40 + 4 weeks on March 4, 2022. At the time of admission, the fetal membranes had not ruptured, and her body temperature was 36.3°C. Abdominal B-mode ultrasound indicated singleton pregnancy and multiple uterine fibroids (the largest was 17 × 8 mm). Chills occurred after oxytocin administration at admission, and her body temperature was 36.7°C. A baby boy was delivered by vaginal delivery, and his birth weight was 4,000 *g*. She had a first-degree perineal tear. After delivery, she was observed for >1 h, and her body temperature increased to 39°C. Emergency blood culture, blood analysis, and procalcitonin test were carried out. Her 4-h postpartum vaginal blood loss was 850 mL. Respiratory disease, hematologic disease, and urinary tract infection were ruled out. Prenatal vaginal discharge evaluation (posterior fornix swab) with a direct microscopic examination found gram-negative or -variable rods, and her Nugent score was 7. Hence, the patient was diagnosed with bacterial vaginosis, and cefoperazone/sulbactam was used for anti-infective treatment. After 5 days of antibiotic treatment, the patient was discharged. The patient appeared well at subsequent visits and seemed to have recovered completely.

### 2.2 Test results

**Figure 1:**
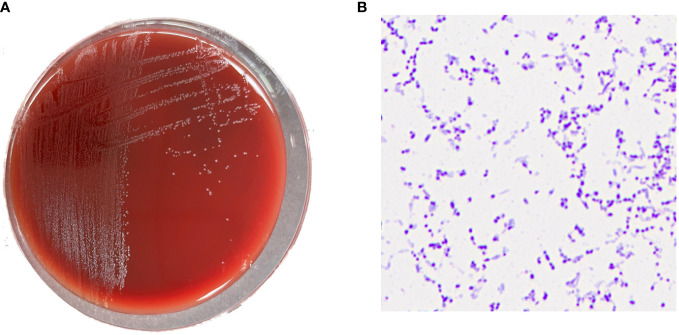
**(A)** Grayish white colonies of *Fannyhessea vaginae* after 48h of culture on blood agar plate under anaerobic conditions; **(B)** Gram staining showing gram-positive (*F*) *vaginae* bacteria appearing as elliptical or short rods.

In bilateral dual-bottle blood culture, the left anaerobic bottle was positive after 50 h of culture. The liquid in the positive blood culture bottle was aspirated and inoculated on blood agar plates, which were cultured in an aerobic environment and an anaerobic environment at 35°C. After 48 h, no bacterial growth was observed in the blood agar plate from the aerobic environment, and small grayish-white colonies could be observed in the plate from the anaerobic environment (**Figure 1A**). The Gram staining smear was positive, and the bacteria were elliptical or short rods in shape (**Figure 1B**). A single colony was selected, matrix-assisted laser desorption/ionization time-of-flight mass spectrometry (Bruker, Germany, MALDI Biotyper 3.1) rapid identification result was *F. vaginae*, and the score was 2.010. Additionally, the 16S rRNA sequence (GenBank accession no. OR287194) analysis also indicated that this bacterium was *F. vaginae*.

### 2.3 Clinical efficacy

**Figure 2:**
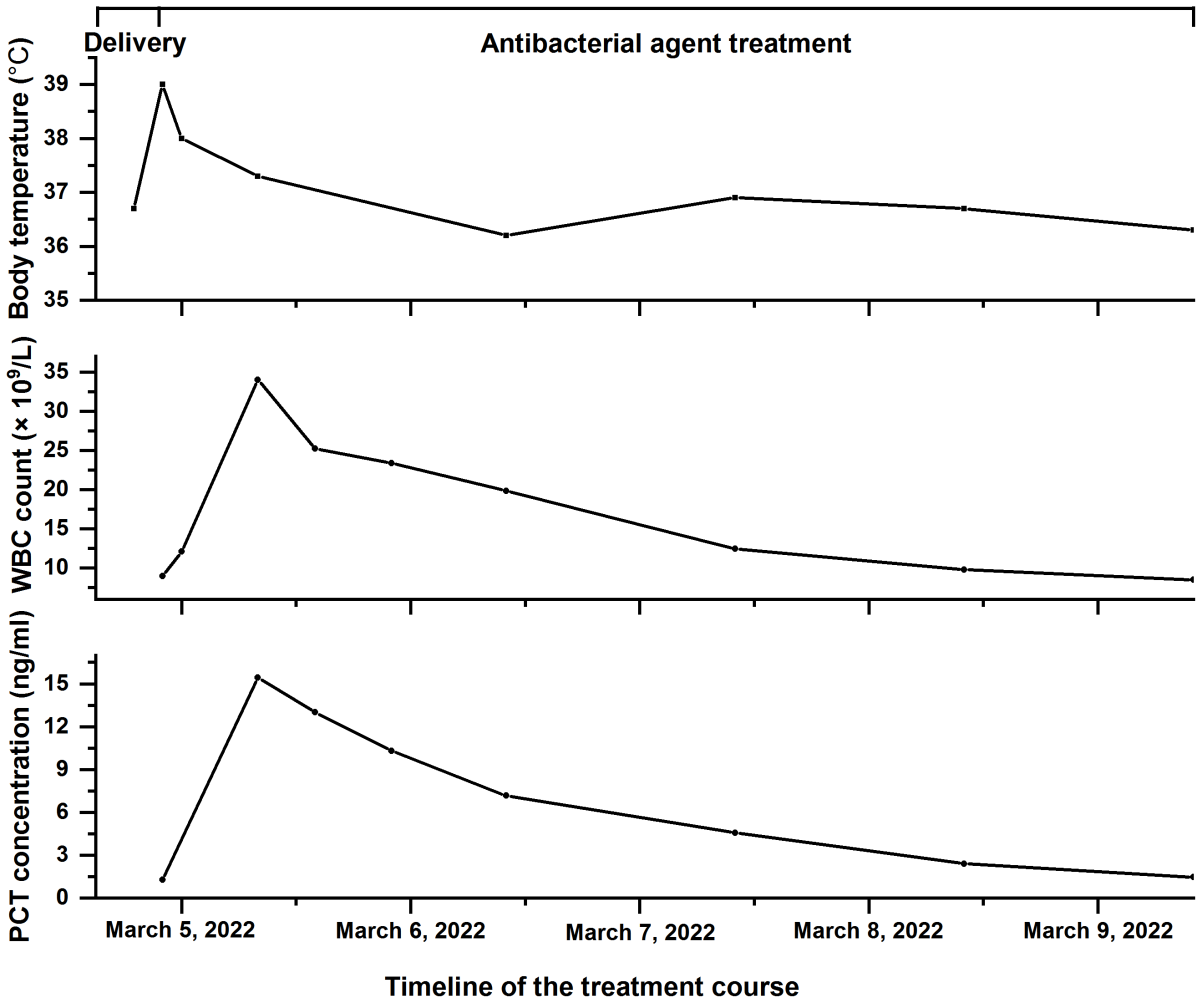
The timeline of the treatment course of the patient with *Fannyhessea vaginae* infection. Body temperature (top), white blood cell (WBC) count (middle), and procalcitonin (PCT) concentration (bottom) during hospitalization.

Cefoperazone/sulbactam was used as an anti-infective treatment when the patient’s white blood cell count peaked at 34 × 10^9^/L. The dose was 3.0 g, intravenous infusion for 8 h. At 5 days postpartum, antibiotic treatment was discontinued. The patient’s white blood cell count and procalcitonin continuously decreased until they returned to normal, and her temperature gradually returned to normal. **Figure 2** displays the variation trend of body temperature, white blood cell count, and procalcitonin concentration.

### 2.4 Drug sensitivity

The minimum inhibitory concentration (MIC) breakpoints for anaerobic bacteria in the Clinical and Laboratory Standards Institute M100-S31 and European Committee on Antimicrobial Susceptibility Testing (Version 10.0) were used as a reference, one McFarland turbidity solution was prepared, and the E test strip was used for the drug sensitivity test. The results are shown in **Table 1**.

**Table 1:** Retrospective antimicrobial testing of the *Fannyhessea vaginae* strain using the *E* test.

Antibacterial agent	MICs (mg/L)	Breakpoints		
		Susceptible	Intermediate	Resistant
Ampicillin	0.094	≤0.5^a^	1^a^	≥2^a^
Clindamycin	0.016	≤2^a^	4^a^	≥8^a^
Imipenem	0.016	≤4^a^	8^a^	≥16^a^
Penicillin	0.19	≤0.5^a^	1^a^	≥2^a^
Metronidazole	>256	≤8^a^	16^a^	≥32^a^
Vancomycin	1	≤2^b^	N/A	≥2^b^
Linezolid	0.38	N/A	N/A	N/A
Ampicillin/sulbactam	0.75	≤8/4^a^	16/8^a^	≥32/16^a^
Piperacillin/tazobactam	0.38	≤16/4^a^	32/4–64/4^a^	≥128/4^a^
Cefoperazone/sulbactam	0.38	N/A	N/A	N/A

a Clinical and Laboratory Standards Institute breakpoints.

b European Committee on Antimicrobial Susceptibility Testing breakpoints.

MIC, minimum inhibitory concentration; N/A, not available.

## 3 Literature review

To the best of our knowledge, there are 10 previously published cases of *F. vaginae* infections, and a list of these cases is summarized in **Table 2**. These *F. vaginae* infections occurred in bloodstream infections (Knoester et al., 2011; Chan et al., 2012; Dauby et al., 2019; Taillandier et al., 2020), tubo-ovarian abscess (Geissdörfer et al., 2003), bacterial vaginosis (Burton et al., 2004), uterine endometritis (Yamagishi et al., 2011), subchorionic hematoma (Jacqmin et al., 2018), endocarditis (Mansell et al., 2018), and prosthetic joint infection (Massa et al., 2022). Our reported case revealed that anaerobic species found in the vaginal tract could cause bacteremia in pregnant women and cefoperazone/sulbactam was effective for antimicrobial treatment of *F. vaginae* infection.

**Table 2:** Summary of 11 reported cases of *Fannyhessea vaginae* infections.

Reference	Age (years)/sex	Country of origin	Disease	Antibiotic susceptibility testing (MICs mg/L)	Antimicrobial treatment	Outcome
Knoester et al. (2011)	40/female (pregnant)	Netherlands	Bacteremia	PEN S (0.094), MTZ R (24), VAN S (1.5), CXM N/A 0.125, CLI S (<0.016)	AMP (1 g administered intravenously four times a day) for 2 weeks	Fetal death and the patient was cured
Chan et al. (2012)	33/female (pregnant)	China	Bacteremia	PEN S (0.25), MTZ R (>256)	AMC (1.2 g administered intravenously three times a day) for 4 days	A neonate was delivered (cesarean section) and the patient was cured
Dauby et al. (2019)	29/female (pregnant)	Belgium	Bacteremia	PEN S (0.03), AMP S (<0.0016), CLI S (0.016), MTZ R (>256)	AMC	A neonate was delivered (vaginal birth) and the patient cured
Taillandier et al. (2020)	57/female	France	Septic shock	MTZ R (N/A), CIP R (N/A), PEN S (N/A), AMC S (N/A), CTX S (N/A), CLI S (N/A), VAN S (N/A)	TZP and GEN	Cured
Geissdörfer et al. (2003)	39/female	Germany	Tubo-ovarian abscess	AMP S (0.032), PEN S (0.125), CXM S (0.19), FOX S (2), IMP S (2), MTZ R(>256)	FOX (2 g administered intravenously three times a day) and MTZ (0.5 g administered intravenously twice a day) for 5 days	Hysterectomy, bilateral salpingectomy, left-sided ovariectomy, appendectomy, and adhesiolysis
Burton et al. (2004)	48/female	Canada	Bacterial vaginosis	N/A	N/A	N/A
Yamagishi et al. (2011)	33/female	Japan	Uterine endometritis	AMP S (0.19), SAM S (0.125), PIP R (1.5), TZP S (1.0), MNO N/A (0.19), MEM S (1.5), IMP S (0.25), ERY N/A (<0.016), CLI S (<0.016), CHL S (1), CFP N/A (3), CSL N/A (1.5), CIP N/A (0.064), OFX N/A (0.19), MTZ R (>256)	AMX (0.5 g administered intravenously three times a day) for 4 days	Cured
Jacqmin et al. (2018)	38/female (pregnant)	Belgium	Subchorionic hematoma	PEN S (0.016), MTZ S (1), AMC S (0.016), CLI S (<0.016)	AMC (1 g administered intravenously four times a day) for 4 days	Termination of pregnancy and the patient cured
Mansell et al. (2018)	18/female	United Kingdom	Endocarditis	N/A	VAN for 4 weeks	Surgical excision of tricuspid valve vegetation
Massa et al. (2022)	77/female	Belgium	Prosthetic joint infection	AMX S (0.047), CIP R (8), CLI S(<0.016), RIF S (<0.002)	TLC and then changed to AMX in combination with RIF on day 19 of admission	Cured
Present case	32/female (pregnant)	China	Bacteremia	AMP S (0.094), CLI S (0.016), IMP S (0.016), PEN S (0.19), MTZ R (>256), VAN S (1), LNZ N/A (0.38), SAM S (0.75), TZP S (0.38), CSL N/A (0.38)	CSL (3 g administered intravenously three times a day) for 5 days	A baby boy was delivered (vaginal birth) and the patient was cured

AMC, amoxicillin/clavulanic acid; AMP, ampicillin; AMX, amoxicillin; CFP, cefoperazone; CHL, chloramphenicol; CIP, ciprofloxacin; CLI, clindamycin; CSL, cefoperazone/sulbactam; CTX, cefotaxim; CXM, cefuroxime; ERY, erythromycin; FOX, cefoxitin; GEN, gentamicin; IMP, imipenem; LNZ, linezolid; MEM, meropenem; MNO, minocycline; MTZ, metronidazole; OFX, ofloxacin; PEN, penicillin; PIP, piperacillin; RIF, rifampicin; SAM, ampicillin/sulbactam; TLC, temocillin; TZP, piperacillin/tazobactam; VAN, vancomycin; MIC, minimal inhibitory concentration; R, resistant; S, susceptible; N/A, not available.

## 4 Discussion

In the last 20–30 years, rapid and precise species-level identification of anaerobes has aided clinicians in providing the best care for their patients, resulting in dramatically lower morbidity and mortality rates and hospital stays (Kovács et al., 2022). However, anaerobic bacteria continue to be among the most neglected and unrecognized pathogens because their cultivation necessitates substantial microbiological experience, and many hospitals (particularly in developing nations) may lack the equipment necessary to achieve anaerobiosis (Nagy et al., 2018). As an anaerobic, *F. vaginae* is found in the normal vaginal microbiota but is increasingly linked to bacterial vaginosis (Mendling et al., 2019). Recently, a prospective investigation linked *F. vaginae* to salpingitis and infertility, indicating this microorganism’s potential pathogenicity (Haggerty et al., 2016).

According to the Nugent score criteria for the identification of bacterial vaginosis via a direct microscopic examination, a Nugent score of 7 can be diagnosed as bacterial vaginosis as in our case report. This vaginal infection case allowed us to hypothesize that an ascending infection of *F. vaginae* from the vagina caused this woman’s bacteremia. Similar ascending bacteremia of *F. vaginae* has also been reported in other cases. For instance, a case reported of a pregnant woman who had *F. vaginae* transferred from her cervix to her uterus during chorionic villus sampling, causing an intrauterine infection that resulted in fetal mortality and bacteremia of the mother (Knoester et al., 2011). Another case described an intrapartum *F. vaginae* bacteremia that occurred spontaneously without any prior surgical trauma to the female genital tract; the case was characterized by an imbalanced vaginal microbiota with the proliferation of *G. vaginalis* and *Candida albicans* (Chan et al., 2012). In addition to bacteremia, other diseases have been documented as a result of ascending *F. vaginae* infections. A 33-year-old woman with bacterial vaginosis was clinically diagnosed with uterine endometritis due to an ascending *F. vaginae* infection (Yamagishi et al., 2011). Similarly, an 18-year-old patient lanced a vaginal cyst herself with a subcutaneous insulin cannula, resulting in infective endocarditis due to an ascending *F. vaginae* infection (Mansell et al., 2018). Therefore, disturbed vaginal microbiota is a significant cause of a variety of diseases in women, and more attention should be given to the vaginal microbiome of female patients with vaginal dysbiosis.

*F. vaginae* infections in pregnant patients have a clinical consequence that affects both the mother and the fetus. Although no maternal deaths had been documented, major morbidities such as the need for extensive surgery and consequent infertility, as well as the emotional toll of losing the fetus, might still occur (**Table 2**). In light of the severe consequences associated with *F. vaginae* infections, appropriate treatment should be initiated once the diagnosis is made.

The European Committee on Antimicrobial Susceptibility Testing states that the sensitivity of anaerobic bacteria to antimicrobial agents is exclusively measured using the MIC technique. However, because commercial automated identification and susceptibility testing systems are not commonly available, treatment of infections caused by these anaerobic microorganisms remains a challenge. Metronidazole is the most commonly used antimicrobial agent against anaerobic bacterial species. However, the results of susceptibility testing on metronidazole for *F. vaginae* are variable because some strains have high MIC values (De Backer et al., 2006). In our reported case, this *F. vaginae* strain was resistant to metronidazole (MIC >256 μg/mL), although the pathogen can be inhibited by low concentrations of clindamycin (MIC of 0.016 μg/mL), another commonly used antimicrobial agent for treating anaerobes.

According to the review of the literature (**Table 2**), penicillin-based antibiotics, such as amoxicillin and piperacillin, were mostly used in the treatment of *F. vaginae* infections in the reported cases. We also performed the susceptibility testing of the *F. vaginae* strain to penicillin-based antibiotics and found that this strain can be inhibited by low concentrations of ampicillin (MIC of 0.094 μg/mL), penicillin (MIC of 0.19 μg/mL), ampicillin/sulbactam (MIC of 0.75 μg/mL), and piperacillin/tazobactam (MIC of 0.38 μg/mL). However, in our case report, based on the understanding that cefoperazone/sulbactam appears in low levels in human milk and are not expected to cause adverse effects in breastfed infants (Matsuda et al., 1985; Lai et al., 2018), the patient was treated empirically with cefoperazone/sulbactam, and the retrospective antimicrobial susceptibility testing confirmed that cefoperazone/sulbactam (MIC of 0.38 μg/mL) was effective at inhibiting pathogen proliferation. In addition to our case report, it has been reported that other cephalosporin antibiotics, such as cefoxitin, are efficacious against *F. vaginae* infections (Geissdörfer et al., 2003). These results demonstrate that cephalosporin antibiotics are also an option for treating *F. vaginae* infections.

## 5 Conclusions

Incidence, morbidity, and death rates due to anaerobic bloodstream infections should be given more attention in patients. As an anaerobic bacterium, *F. vaginae* is found in normal vaginal microbiota; however, under certain conditions, it may cause life-threatening infections. If a patient with bacterial vaginosis is febrile and exhibits bloodstream infection symptoms during the postpartum period, it is vital to be mindful of bacterial vaginosis associated anaerobic species such as *F. vaginae* and to adapt the empirical therapy, as was the case here.

## Data availability statement

The datasets presented in this article are not readily available because of ethical/privacy restrictions. Requests to access the datasets should be directed to the corresponding author.

## Ethics statement

The study was conducted in accordance with the Declaration of Helsinki and approved by the Institutional Review Board (protocol code 2023-1-026). Written informed consent was obtained from the individual(s) for the publication of any potentially identifiable images or data included in this article. Written informed consent was obtained from the participant/ patient(s) for the publication of this case report.

## Author contributions

PL: Writing – original draft. LW and RL: Data curation. XC: Writing – review & editing.
